# RSV Vaccination Programme for Older Adults: A Scotland-Wide Study on RSVpreF Vaccine Safety

**DOI:** 10.3390/vaccines13111088

**Published:** 2025-10-24

**Authors:** Lucy A. Cullen, Cheryl L. Gibbons, Ting Shi, Taimoor Hasan, Christopher Sullivan, Kimberly Marsh, Chris Robertson, Joanne Claire Cameron, Sam Ghebrehewet

**Affiliations:** 1 Public Health Scotland, Edinburgh EH12 9EB, UKclaire.cameron2@phs.scot (J.C.C.);; 2 Usher Institute, University of Edinburgh, Edinburgh EH16 4UX, UK; 3 Department of Mathematics and Statistics, University of Strathclyde, Glasgow G1 1XH, UK

**Keywords:** vaccine safety, RSV vaccination, vaccine confidence, immunisation, adverse events

## Abstract

Background/Objectives: Respiratory Syncytial Virus (RSV) is a common respiratory tract infection that accounts for significant morbidity and mortality, particularly among older adults. From 1 August 2024, the bivalent RSVpreF vaccine (Abrysvo^®^) was introduced in Scotland for eligible older adults. While clinical trials and post-licensure studies showed a good safety profile of the vaccine, post-marketing observational studies in the United States reported a small increased risk of Guillain–Barré Syndrome (GBS) among older adult populations. Methods: We conducted observed versus expected (OE) and self-controlled case series (SCCS) analyses to retrospectively monitor the incidence of hospital admission for 39 conditions, including GBS. This was undertaken in post-RSV-vaccination periods from 1 August to 31 December 2024, among eligible adults aged 74 to 80 years in Scotland. Results: Observed versus expected analyses identified an increased risk of hospitalisation with GBS in the 1–28-day post-vaccination period. From SCCS analyses, six conditions showed an increased risk in post-vaccination periods (acute coronary syndrome, acute myocardial infarction, acute renal failure, GBS, heart failure and stroke (haemorrhagic)). After temporal adjustment, only GBS remained significant. All 10 hospitalised GBS cases occurred in the 10 to 16 days post-vaccination. Excess risk of GBS was estimated to be 46.1 cases per one million doses. Conclusions: Study results indicated a good safety profile of the RSV vaccine for older eligible adults aged 75 to 79 years, although a small increased risk of GBS was identified in both analyses. Excess risk levels of GBS from vaccination align with other studies.

## 1. Introduction

Respiratory Syncytial Virus (RSV) is a common infection that usually causes mild respiratory infection in adults and children. It can, however, cause severe lower respiratory infection (LRTI) in infants and older adults, accounting for significant morbidity and mortality [1,2]. In particular, older adults with comorbidities are especially vulnerable to severe outcomes of infection [3]. The RSV bivalent prefusion F vaccine (RSVpreF) developed by Pfizer, known as Abrysvo^®^, was licenced for use in adults over 60 years of age in the United States (US), Europe and the UK in May, August and November 2023, respectively [4,5,6].

A recently published integrated analysis of eight clinical trials reported a favourable safety profile of the RSVpreF vaccination [7]. Among 18- to 59-year-olds, no increased risk of serious adverse events in RSVpreF recipients was identified compared with placebo. Similarly, no evident safety concerns were identified from a clinical trial conducted in adults aged 60 and over. However, three serious adverse events that occurred in the older adult population were considered possibly related to RSVpreF and were recommended for further monitoring. These included two Guillain–Barré Syndrome (GBS) cases (including Miller Fisher Syndrome, a variant of GBS) and a delayed allergic reaction [8]. The trial also found the vaccine to be highly effective at preventing RSV-associated LRTIs in older adults. No other serious adverse events were identified in clinical trials.

Clinical trials are often limited in sample size and heterogeneity, and therefore, may be too small to assess rarer outcomes or may not reflect the entire population. Ongoing surveillance following the roll out of a national programme is essential to detect rare adverse events and monitor vaccine safety in the population. Real-world data from healthcare databases are useful for identifying and evaluating potential safety signals to complement and expand upon evidence from clinical trials. In post-licensure observed versus expected (OE) and self-controlled case series (SCCS) analyses in the United States (US), a statistically significant elevated risk of GBS was reported in adults aged 65 years and older. This risk was no longer statistically significant after adjusting for the positive-predictive value of case identification [9]. A larger observational study of over 3.2 million adults aged 60 and over in the US also identified a statistically significant increased risk of GBS following RSVpreF vaccination, corresponding to an excess risk of 18.2 cases per one million doses [10]. These findings warrant continued real-world safety monitoring in all countries where the vaccine is being administered.

The RSVpreF vaccine was first offered in Scotland to older adults turning 75 years old between 1 August 2024 and 31 July 2025, alongside a catch-up campaign for those aged 75 to 79 years old. The vaccine schedule comprises a single dose and is currently a one-off offer unlike seasonal influenza [1]. In Scotland, the offer began in late summer to accommodate winter (influenza and COVID-19) vaccination programmes and to allow time for immunity to build prior to the RSV season, which is typically earlier than the influenza season. There was also some ongoing opportunistic vaccination and potential for mop-ups outside of the winter programme.

This study aimed to assess the potential for elevated risk of clinical adverse events following introduction of the RSV vaccination in the older adult Scottish population using real-world linked healthcare data.

## 2. Materials and Methods

### 2.1. Study Design

Two retrospective observational analyses were designed to carry out the following:

1. Estimate the OE ratios of pre-specified adverse events of special interest (AESI) following RSV vaccination of older adults, using an OE design.
2. Estimate the relative incidence of AESI within pre-defined risk periods following RSV vaccination of older adults compared with pre-vaccination risk periods, using a SCCS design.

### 2.2. Data Sources

The overall study population was identified from individuals aged 74 to 80 years in receipt of an RSV vaccination in Scotland from 1 August 2024 to 31 December 2024. Patient-level RSV vaccination data are recorded using the Turas Vaccine Management Tool (VMT) [11], a web-based tool for healthcare staff in Scotland to record real-time patient vaccination data at the point of care. These data flow to the National Clinical Data Store (NCDS), from where data were extracted on 3 March 2025. For the primary OE analysis, individuals were only included if they were aged 75 to 79 years old at the time of vaccination to match the age breakdowns available in the National Records of Scotland (NRS) mid-year population estimates [12] for years from 2015 to 2023. For the secondary SCCS analysis, all individuals vaccinated as part of the older adult programme were included regardless of their age (i.e., may include individuals aged 74 to 80 years inclusive).

Hospitalisation data were extracted from the Scottish Morbidity Records 01 (SMR01) dataset [13], which includes inpatient data from all hospitals in Scotland. Mortality data were sourced from NRS [14].

### 2.3. Exposure of Interest

Receipt of one dose of RSV vaccine (RSVpreF) in Scotland, as entered in the VMT and identified through the NCDS.

### 2.4. Outcomes of Interest

Thirty-nine AESI (Supplementary Table S1) were included, primarily based on those monitored in safety surveillance of the COVID-19 vaccinations [15]. Additional outcomes were identified from clinical trials for RSV, yellow card reports to the Medicines and Healthcare products Regulatory Agency (MHRA), and those suggested by the PROMISE (Preparing for RSV Immunisation and Surveillance in Europe) consortium [16]. AESI were identified from SMR01 hospitalisation records using International Classification of Diseases-10th Revision codes (ICD-10) [17] in any “main_condition” or “other_condition” of any episode. Episodes were aggregated to patient stay level with the date of an event taken as the first date of hospital admission for the stay, regardless of the episode an AESI diagnosis was recorded in.

For the OE analyses, a clean window was applied to differentiate between new (incident) cases and ongoing or recurring episodes for each AESI. Specifically, any subsequent hospitalisations for the same condition occurring within a pre-defined clean window period were assumed to be related [15]. Risk periods were the hypothesised post-vaccination time frames with increased likelihood of AESI occurring following exposure. Both clean windows and risk periods were identified from the literature where possible. If a specific risk period was not known, several adjacent risk periods were included with the aim of capturing the true risk period. Risk periods were also combined to help avoid intervals with zero observations. All AESI outcomes and their associated definitions were reviewed for their suitability by clinicians.

Age and sex adjustments were not made due to the narrow demographic eligible for vaccination.

### 2.5. Statistical Analyses

#### 2.5.1. Observed Versus Expected (OE)

**Figure 1:**
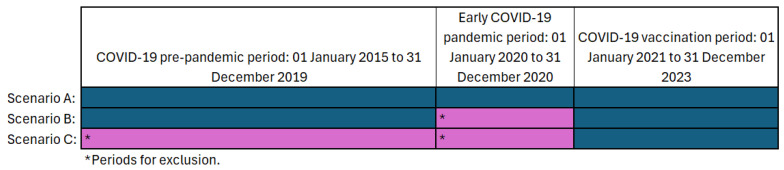
Illustration of the background rate periods used for each AESI based on changes to the Incidence Rates (IRs) and 95% confidence intervals (CI) throughout the period 1 January 2015 to 31 December 2023.

OE analyses retrospectively compared background (pre-RSV-vaccination implementation) incidence rates (IRs) with post-RSV-vaccination implementation IRs of AESI. A post-vaccination risk period of 1–28 days (where day 0 represented the day of vaccination) was used for all AESI except seizures, for which 0–6 days was used due to the increased likelihood of rapid and proximal onset. Background IRs of AESI events (the number of AESI cases per 100,000 population) were calculated using the same methodology outlined previously for COVID-19 vaccine safety surveillance [15]. Background IRs used to calculate expected post-RSV-vaccination implementation IRs should reflect the IRs at the time of RSV vaccination implementation as closely as possible. Where possible, the total period from 1 January 2015 to 31 December 2023 was used to calculate background IRs. However, it is recognised that the early COVID-19 pandemic (1 January 2020 to 31 December 2020) and COVID-19 vaccination periods (1 January 2021 to 31 December 2023) may have impacted IRs [15]. Therefore, for each AESI, three background rate periods were considered and their IRs were defined as different in one period compared to another (outliers) where the IR and 95% confidence intervals (CI) were higher or lower than the comparison period with no overlap. Figure 1 illustrates the three background rate periods used based on these scenarios. Where all the IRs overlapped with the early COVID-19 vaccination period (scenario A), the IRs for the total period were used as the background rate period. Where IRs in the early COVID-19 pandemic period were the only outlier (scenario B), the early COVID-19 pandemic period was removed from the total background rate period. Where the pre-COVID-19 pandemic period differed from the most recent period (COVID-19 vaccination), the assumption was made that the early COVID-19 pandemic had an impact on AESI rates, and only the most recent COVID-19 vaccination period was used (scenario C).

Possible safety signals were identified where the OE ratio and 95% CI were greater than 1.0 (i.e., the observed rate was significantly higher than the expected rate) [18], and there were three or more observed events in the post-vaccination risk period. Detailed methods for the OE analyses are available in Supplementary Table S2.

#### 2.5.2. Self-Controlled Case Series (SCCS)

SCCS retrospectively compared the rate of hospitalisations containing a recorded AESI in post-vaccination risk period(s) with the rates during unexposed pre-vaccination control periods for these individuals using conditional logistic regression. All hospital stays in the risk periods were included to study both incident cases and exacerbations of existing conditions. An incident rate ratio (IRR) and 99% CI greater than 1.0 indicated an increased rate of hospitalisation following vaccination and were deemed significant where *p* < 0.01, prompting further investigation of a potential safety signal. A *p*-value < 0.01 was likely to identify more signals than alternative approaches used to adjust significance for multiple testing (e.g., Bonferroni method or False Discovery Rate method) but was chosen for pragmatic purposes and to increase the sensitivity of the surveillance conducted. Where potential signals were identified, a stringent approach was taken to investigate the hospitalised individuals through characterisation (see Section 2.5.3 below).

SCCS was conducted on AESI where three or more hospitalisations occurred in the post-vaccination risk period(s). A minimum of three hospitalisations was specified to increase study power and reduce the likelihood that events in the post-vaccination period occurred as rare one-off events coincidental to vaccination. Detailed methods for the SCCS analysis are presented in Supplementary Table S3.

Where there were enough hospitalisations to reach the threshold for conducting SCCS, the analysis for respiratory failure and vasculitis included a temporal stratification of calendar time periods (28 days) to account for seasonal variation that is known from previous analyses to exist in hospitalisations for these AESI in Scotland [15]. Monthly trends of IRs from background rate periods calculated in the OE analysis were reviewed to identify any additional AESI with seasonal variation in the 75–79-year-old age group among those AESI with signals. Searches were also conducted to check for evidence of seasonality for signalling AESI reported in the literature. SCCS for these AESI was then repeated including the temporal stratification of calendar time (28 or 56 days) in the model to help account for any variations identified that may have affected the results.

Prospectively, the potential power for the SCCS analysis was calculated using the samplesize() function from the SCCS R package version 1.7, inputting different IRR and risk periods with a 0.01 significance level.

#### 2.5.3. Characterisation of Signals

AESI hospitalisations that contributed to potential signals identified from the OE and SCCS analyses were interrogated further to characterise the cases. This characterisation was conducted to identify any patterns or frequencies in hospitalisation, which could provide insight into their association with RSV vaccination that may warrant further investigation and aid our understanding of their likelihood of being flagged as a signal by chance.

#### 2.5.4. Attributable Risk

Where an AESI was consistently identified with a signal across the analyses, the attributable risk was calculated using the following formula:

Attributable cases per 1 million dose={[(IRR − 1)/IRR] × number of events during risk period}/number of eligible vaccinations×1,000,000

This gave an estimate of the excess risk of the AESI in the post-vaccination period [10].

## 3. Results

### 3.1. Descriptive Statistics

Between 1 August 2024 and 31 December 2024, there were 204,140 adults aged 74–80 years vaccinated with the RSVpreF vaccine. Of these, 35,475 were aged either 74 years or 80 years at the time of vaccination and were therefore excluded from the OE analysis (Table 1). As of 3 March 2025, this equates to an uptake of 72.1% of 75–79-year-olds in Scotland vaccinated by 31 December 2024, based on 2023 mid-year population estimates.

### 3.2. Observed Versus Expected (OE Analysis)

The background rate periods used for each AESI are outlined in Table 2, based on IR comparisons between the COVID-19 pre-pandemic, early COVID-19 pandemic and COVID-19 vaccination periods (Figure 1).

OE results identified two of the 39 AESI with higher numbers of hospital admissions in the 1–28 day post-vaccination period compared with expected: GBS with an OE ratio of 12.1 (5.5–23.0 95% CI), and Optic Neuritis with an OE ratio of 11.4 (1.4–41.1 95% CI) (Table 2). Of these, only GBS met the threshold of three observed events or more in the risk period required to constitute a safety signal. Nine hospitalisations with a GBS diagnosis were observed in the 1–28 day post-vaccination period compared to the expected 0.74 hospitalisations. Characterisation of these nine GBS post-vaccination cases found five were male (56.0%). Cases were admitted to hospital with a mean of 12.6 days post-vaccination (range: 10–16 days), and GBS was diagnosed as the main condition within the first episode on admission to hospital for all nine individuals (100.0%).

### 3.3. Self-Controlled Case Series Analysis (SCCS)

**Figure 2:**
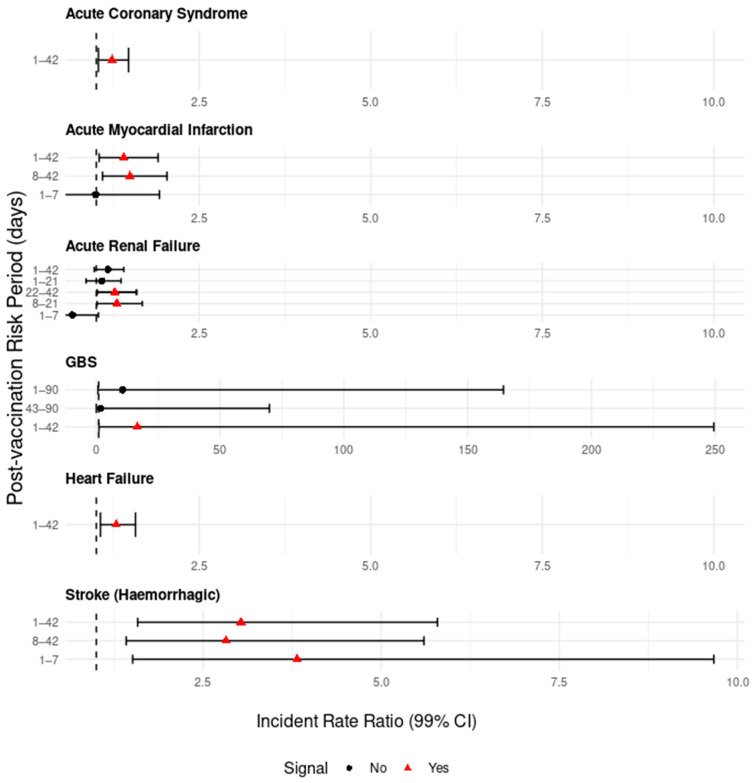
SCCS results for all AESI with a signal identified in at least one post-RSV-vaccination risk period, among 75–79-year-olds in Scotland from 1 August to 31 December 2024. Incident rate ratios (IRR; 99% confidence intervals) are shown for acute coronary syndrome, acute myocardial infarction, acute renal failure, Guillain–Barré syndrome (GBS), heart failure and haemorrhagic stroke in post-vaccination risk periods. Signals are indicated with a red triangle where SCCS analysis identified a statistically significant increase in the rate of hospitalisations following vaccination. The black dotted line indicates an IRR of 1.0.

SCCS analysis was not conducted for 13 AESI that had fewer than three hospital admissions in their longest pre-defined post-vaccination risk period (acute and subacute hepatic failure, anaphylactic shock, autoimmune thyroiditis, chronic fatigue syndrome, demyelination, disseminated intravascular coagulation, encephalitis including acute disseminated encephalomyelitis (ADEM), intracranial venous thrombosis, narcolepsy, neuromyelitis optica, subsequent myocardial infarction, transverse myelitis and vasculitis). The remaining 26 AESI had three or more hospital admissions in the longest post-vaccination risk period of interest (Supplementary Table S3). Of those, SCCS identified a significant increase (signal) in the rate of hospital stays in at least one post-vaccination risk period for six AESI: GBS, acute coronary syndrome, acute myocardial infarction (AMI), heart failure, stroke (haemorrhagic) and acute renal failure (Table 3, Figure 2).

#### 3.3.1. Nervous System Signals

For GBS, a signal was identified from 10 hospital stays in the 1–42 day post-vaccination period (IRR: 16.6, 99% CI 1.1–249.4). Characterisation of the 10 individuals contributing to this signal found five (50.0%) were male with a mean age of 76.5 years (range: 74–79). This equates to a 1.1 times higher incidence in males at 5.1 cases per million doses among males versus 4.7 cases per one million doses among females. Mean time from vaccination to hospital admission was 12.9 days (range: 10–16 days), and GBS was the main condition on admission to hospital for all patients (100.0%). The increased rate of GBS was not seen in the 43–90 days (IRR: 1.8, 99% CI 0.04–69.9) or 1–90 days post-vaccination (IRR: 10.6, 99% CI 0.7–164.5). The study was powered to detect a 10-fold increase in GBS with 65.0% power for the 90-day risk period.

#### 3.3.2. Circulatory System Signals

The signals for AMI were identified from 142 hospital stays within the overall 1–42 day period (IRR: 1.4, 99% CI 1.0–1.9), with the majority (125, 80.0%) occurring in the 8–42 day period (IRR: 1.5, 99% CI 1.1–2.0). There was no signal in the 1–7 day risk period (IRR: 1.0, 99% CI 0.5–1.9). The mean age of the 138 individuals (142 stays) was 76.7 years (range: 74–80) with a mean of 21.5 days (range: 1–42) from vaccination to hospitalisation. AMI was the main condition on admission to hospital for the majority (118, 85.5%). The study was powered to detect a 1.4-fold increase in AMI with 50.0% power for the 42-day risk period.

Acute coronary syndrome signalled from 398 hospital stays in the 1–42 day risk period (IRR: 1.2, 99% CI 1.0–1.5). Of the 367 hospitalised individuals, 227 (61.9%) were male with a mean age of 76.7 years (range: 74–80). The mean number of days from vaccination to hospital admission was 21.9 (range: 1–42), and 178 (48.5%) had acute coronary syndrome as their main condition on admission to hospital. The study was powered to detect a 1.2-fold increase in acute coronary syndrome with 45.0% power for the 42-day risk period.

There were 318 hospital stays with heart failure in the 1–42 days post-vaccination risk period (IRR: 1.3, 99% CI 1.1–1.6). This signal came from 303 individuals (173, 57.1% male) with a mean age of 76.8 (range: 74–80). There was a mean of 22.3 days (range: 1–42) between vaccination and hospitalisation, and heart failure was the main condition on admission to hospital for 70 (23.1%) patients. The study was powered to detect a 1.2-fold increase in heart failure with 35.0% power for the 42-day risk period.

Signals for stroke (haemorrhagic) were identified across all post-vaccination risk periods: 1–7 days (IRR: 3.8, 99% CI 1.5–9.7), 8–42 days (IRR: 2.8 99% CI 1.4–5.6) and 1–42 days overall (IRR: 3.0, 99% CI 1.6–5.8). Of the 45 individuals (46 hospitalisations), the mean age was 77.0 years (range: 74–80) and 29 were male (64.4%). Mean time from vaccination to admission was 18.6 days (range: 1–42), with 33 individuals (73.3%) with stroke as their main condition on admission. The study was powered to detect a three-fold increase in stroke (haemorrhagic) with 90.0% power for the 42-day risk period.

#### 3.3.3. Urinary System Signals

Signals for acute renal failure were identified in the 8–21 days (IRR: 1.3, 99% CI 1.0–1.7) and 22–42 days post-vaccination (IRR: 1.3, 99% CI 1.0–1.6). This increase was not observed in the 1–7 day risk period (IRR: 0.7, 99% CI 0.4–1.0), 1–21 day risk period (IRR: 1.1, 99% CI 0.9–1.4) or the overall 1–42 day risk period (IRR: 1.2, 99% CI 1.0–1.4). Characterisation of the 316 individuals (322 hospitalisations) within the 8–42 day post-vaccination period found they had a mean age of 76.7 years (range: 74–80) and 166 were male (52.5%). There was a mean of 24.4 days (range: 8–42) from vaccination to hospitalisation and 41 (13.0%) had acute renal failure as their main condition on admission. The study was powered to detect a 1.2-fold increase in acute renal failure with 35.0% power for the 42-day risk period.

#### 3.3.4. Seasonal Adjustments—Temporal Stratification of Calendar Time

Review of the background IRs in Scotland for hospitalisations with signalling AESI among 75–79-year-olds did not identify any marked seasonal trends for AMI, acute coronary syndrome or heart failure. However, there is evidence from the literature that these cardiovascular conditions have higher incidence rates in winter months [19], which coincide with the post-vaccination risk periods. In addition, marginal seasonality was identified from the total background rate period IRs for stroke (haemorrhagic) and acute renal failure, with highest IRs in winter months. This agreed with peer-reviewed research evidencing higher incidence of acute kidney injury in winter months [20] and higher hospitalisation rates for intracranial haemorrhage in winter months in Scotland [21]. Finally, there was no evidence of seasonality from background IRs for GBS, but evidence from the literature is somewhat mixed, with higher incidence rates in winter months occurring, depending on geographical location [22].

**Figure 3:**
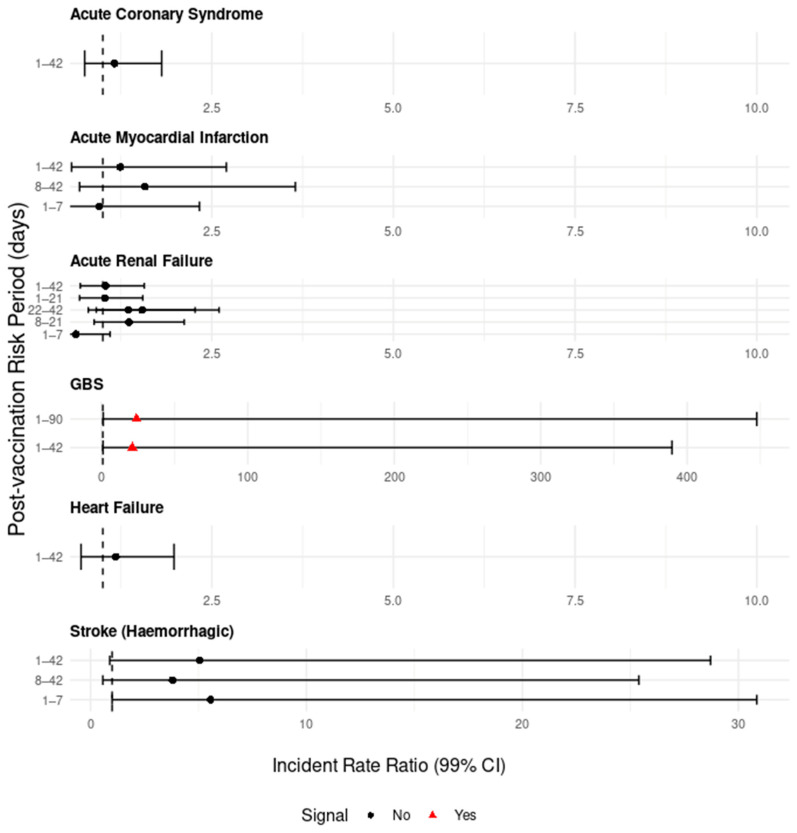
SCCS results with seasonal adjustments (temporal stratification of calendar time) for AESI that had a signal in at least one post-RSV-vaccination risk period in the unadjusted results, 75–79-year-olds in Scotland. Incident rate ratios (IRR; 99% confidence intervals) are shown for acute coronary syndrome, acute myocardial infarction, acute renal failure, Guillain–Barré syndrome (GBS), heart failure and haemorrhagic stroke, in post-vaccination risk periods. Signals are indicated with a red triangle where SCCS analysis identified a statistically significant increase in the rate of hospitalisations following vaccination. The black dotted line indicates an IRR of 1.0.

Given that there was some evidence of seasonality, in a sensitivity analysis, the SCCS model was re-run for all six signalling AESI after including a temporal stratification of calendar time (56 days for GBS to account for small numbers, 28 days for the remaining AESI). By adjusting for seasonality, this analysis found no significant increase in post-vaccination hospitalisations for acute coronary syndrome (1–42 day IRR: 1.2 99% CI 0.8–1.8), AMI (1–42 day IRR: 1.2 99% CI 0.6–2.7), acute renal failure (1–42 day IRR: 1.5 99% CI 0.9–2.5), heart failure (1–42 day IRR: 1.2 99% CI 0.7–2.0) or stroke (haemorrhagic) (1–42 day IRR: 5.1 99% CI 0.9–28.7). However, the signal for GBS remained, and there were significant increases in hospitalisations in both the 1–42 day (IRR: 21.2 99% CI 1.2–389.6) and 1–90 day risk periods (IRR: 24.0, 99% CI 1.3–447.5) (Table 4, Figure 3).

1. Temporal stratification of 28 days was used for all AESI except for GBS, which used 56 days. This was due to small numbers.
2. The 43–90 day risk period for GBS is not shown due to small numbers producing an infinite upper 99% CI value (Table 4).

#### 3.3.5. Attributable Risk for GBS

Given the statistically significant increased rate of GBS across all analyses conducted, the attributable risk for the 1–42 day post-vaccination risk period was calculated. Where the IRR = 16.6, number of events = 10 and the number of eligible vaccinations = 204,140, this gave an estimated attributable risk of 46.1 cases per one million doses of RSVpreF.

## 4. Discussion

The first year of the RSV vaccination programme in Scotland successfully delivered vaccines to over 70% of older adults during the study period [23]. An early study of the population level impact showed a 62% reduction in hospitalisations among the eligible age groups in Scotland [24].

In addition to the demonstrable impact of effectiveness in Scotland, results from this study demonstrated a good safety profile of the RSVpreF vaccine. In the analyses, the majority of AESI showed a similar or reduced number of hospitalisations when compared to those that were expected. As in other post-marketing studies, we also identified an increased risk of GBS hospitalisations across both OE and SCCS study designs. This risk remained after adjusting for potential seasonal differences in GBS incidence.

GBS is a rare neurological disease whereby the immune system mistakenly damages the peripheral nervous system, resulting in rapid-onset muscle weakness, and is more common in older adults and in men [25]. Previous reports of GBS following other vaccinations are very rare, including following influenza vaccination, depending on the flu season [26]. In particular, the swine flu vaccine used in 1976–77 was associated with an excess of 10 GBS cases per million doses [26]. The incidence of GBS post-influenza vaccination has since been estimated at one or two cases per million doses, if a causal relationship exists at all [27].

Our estimate of 46.1 cases of GBS per one million doses of RSVpreF is higher than the 9.0 and 18.2 cases per one million doses estimated in US post-marketing studies, respectively [9,10]. This difference in identified risk may reflect the different study age groups included, as the US studies included younger cohorts of adults aged 60 and 65 years and over. It is also worth noting the use of a 43–90 day post-vaccination control period in the other studies.

Although the risk of GBS had been reflected in patient information leaflets for the RSVpreF vaccine already, preliminary results from our current study have been used to update the risk to 15–25 cases per one million doses of RSVpreF in Scotland and England combined [28]. Characterisation of the GBS cases in our study found that all hospitalisations contributing to signals from the 1–42 day risk period occurred within the 10–16 days post-vaccination, indicating this period as having the greatest risk. The finding that half of the hospitalisations occurred in males, despite vaccinating more females, aligns with the risk of GBS being greater in men [29].

Increased hospitalisations post-vaccination for some circulatory and urinary tract system conditions were also identified from SCCS analysis. None of these signals were identified in the OE analysis, with some conversely finding lower than expected (95% upper CIs less than 1.0) hospitalisations in the post-vaccination period (acute coronary syndrome, heart failure and acute renal failure). This may be due to the healthy vaccinee bias, whereby people delay vaccination due to ill health, impacting the shorter 28-day post-vaccination period included in the OE analysis. In addition, when adjusting for seasonality in the SCCS model, any increases in rates were no longer significant, suggesting that the increased rates may have been coincidental to vaccination or higher rates of viral respiratory infections during the winter season. Evidence for seasonality is supported in the literature for these conditions, with higher incidence commonly found in winter months [19,20,21,30], potentially coinciding with the post-vaccination periods included in the study. There is also a relationship between colder weather spells and higher incidence of circulatory conditions independent of season [31]. It should be noted that none of these conditions were reported as safety signals following the RSVpreF vaccination in older adults in any post-licensure safety studies conducted in the US, or from any clinical trials.

The study had a number of limitations. Notably, most of the SCCS analyses were limited in power due to small numbers of people hospitalised for the AESI in baseline and post-vaccination risk periods. This is typically the case for rare events. Use of a pre-vaccination control period may have contributed to lower hospital admissions in baseline periods if people delayed vaccination due to illness. As an observational study, this was outside the control of the analyses, but nevertheless it is a limitation. Additional monitoring of potential AESI once a larger sample size is available is required. Our identification of cases may also have missed AESI occurrences that did not require inpatient hospitalisation. Therefore, milder instances related to vaccination would not have been picked up. In the case of more serious conditions, for example GBS, it is likely that most cases would seek medical attention and be admitted to hospital. Furthermore, the potential risk of GBS in this older age group has already been identified in clinical trials, making it less likely that cases would go undetected. Although a diagnosis of GBS requires complex clinical and laboratory evaluations, we were unable to review patient case notes and validate diagnoses. Therefore, we relied on clinical coding of conditions in hospital records, and as a result, some misclassification errors cannot be ruled out. Although clinically reviewed, the chosen post-vaccination risk periods are also subject to error and may not have reflected the true risk periods. For example, we included the 43–90 days post-vaccination as a risk period for GBS, where others have instead used the same period as their baseline period for SCCS analysis [10]. We did not identify any significant change in incidence of GBS in this period compared with our control period. The use of adjacent risk periods is likely to have reduced the likelihood of missing true risk periods.

It should also be noted that there were higher rates of influenza activity, particularly among older adults, seen during the 2024/2025 winter season in Scotland than observed in previous years [32]. Influenza infection is associated with GBS [33], with studies reporting as much as an 18-fold increase following infection [34], as well as cardiovascular complications, including myocardial infarction [30,35]. High infection rates may therefore have contributed to higher IRs of certain AESI in post-vaccination periods. This was not investigated fully as it was out of scope for this study. However, the majority of older adult RSV vaccinations were delivered by the end of September, whereas extraordinary levels of influenza activity were not seen until more than eight weeks (56 days) later, at the beginning of December, which was beyond the risk period end for most AESI. Therefore, the impact of this potential confounding is likely to be small. Finally, statistical association between vaccination and AESI hospitalisations, identified as signals, does not confirm a causal relationship, and there may be other reasons behind apparent signals or inflation of magnitude of signal.

This study also had several strengths. This was a national study using large, linked data sources of individual patient records. A comprehensive list of 39 AESI were monitored in the analyses based on those conditions most likely to present a risk following vaccination, as identified from previous safety surveillance for respiratory vaccinations, reports to the MHRA and proposals from the literature. Use of the SCCS method eliminated time-invariant confounding bias, and conducting this alongside an OE study allowed for AESI to be evaluated using two complementary but different approaches. This provides further confidence where consistent results were identified across both methodologies. In addition, our findings are consistent with the safety findings identified elsewhere to date.

## 5. Conclusions

Our study adds to the growing literature of evidence providing reassurance relating to the RSV vaccine (RSVpreF) safety for older adults. This study found an increased risk of GBS, which is consistent with other studies. Here, we also identified a narrow GBS risk period from 10 to 16 days post-vaccination, with slightly higher incidence (and therefore risk) among males, and an overall excess risk of 46.1 cases per one million doses of RSVpreF. The risk of GBS should be closely monitored to ensure safety and confidence in the vaccine is maintained and increase awareness among health professionals to aid notification, reporting and early identification of symptoms among affected individuals. Potential signals for other urinary and circulatory conditions were also identified, but findings were inconsistent across our analyses and are likely to be coincidental findings. Signals for these adverse events have not been identified elsewhere. Future surveillance could also look to include GP data. The analyses conducted provides a robust methodology that can be adapted for other vaccination programmes as required. As the RSV vaccination programme continues to develop and we accumulate additional vaccination data from future seasons, there is potential for these analyses to be replicated.

## Figures and Tables

**Table 1:** Demographics of the vaccinated populations included in the study.

Statistical Method	Vaccinated Population (*n*) to 31 December 2024	Male Sex (*n*, %)	Mean Age in Years (Min–Max)
Observed vs. Expected	168,665 ^1^	80,949 (48.0%)	76.8 (75–79)
Self-controlled Case Series	204,140 ^1^	98,123 (48.1%)	76.4 (74–80)

^1^ Includes people who died or moved from Scotland after their vaccination so may differ from RSV vaccine uptake data reported elsewhere.

**Table 2:** Observed versus expected results for all AESI among 75–79-year-olds in receipt of an RSVpreF vaccination in Scotland, 1 August to 31 December 2024.

Adverse Event of Special Interest	Observed Events in Risk Period ^1^	Expected Events in Risk Period ^1^	OE Ratio (95% CI)
Acute and Subacute Hepatic Failure	1	0.99	1.01 (0.03–5.65)
Acute Coronary Syndrome ^b^	155	188.19	0.82 (0.70–0.96)
Acute Myocardial Infarction ^b^	78	74.91	1.04 (0.82–1.30)
Acute Renal Failure ^b^	176	238.73	0.74 (0.63–0.85)
Anaphylactic Shock ^a^	0	0.03	0.00 (0.00–121.58)
Angina Pectoris ^b^	80	112.46	0.71 (0.56–0.89)
Atrial Fibrillation ^a^	251	330.42	0.76 (0.67–0.86)
Autoimmune Thyroiditis ^a^	0	0.51	0.00 (0.00–7.26)
Bulbar Palsy ^a^	4	3.39	1.18 (0.32–3.02)
Chronic Fatigue Syndrome ^a^	0	0.54	0.00 (0.00–6.89)
Demyelination ^a^	0	0.10	0.00 (0.00–37.64)
Disseminated Intravascular Coagulation ^a^	0	0.40	0.00 (0.00–9.18)
Deep Vein Thrombosis and Pulmonary Embolism ^b^	25	48.35	0.52 (0.33–0.76)
Encephalitis including ADEM ^a^	0	0.74	0.00 (0.00–4.98)
Facial Nerve Disorders inc. Bell’s Palsy ^b^	2	3.66	0.55 (0.07–1.98)
Fibromyalgia ^b^	6	8.86	0.68 (0.25–1.47)
**GBS ^2^** ^a^	**9**	**0.74**	**12.1 (5.53–22.96)**
Heart Failure ^c^	114	146.91	0.18 (0.64–0.93)
Intracranial Venous Thrombosis ^a^	0	0.35	0.00 (0.00–10.52)
Lymphadenopathy ^a^	8	13.17	0.61 (0.26–1.2)
Multiple Sclerosis ^a^	8	6.09	1.31 (0.57–2.59)
Myasthenia Gravis ^a^	2	2.27	0.88 (0.11–3.18)
Myocarditis and Pericarditis ^b^	0	2.51	0.00 (0.00–1.47)
Narcolepsy ^a^	0	0.03	0.00 (0.00–112.75)
Neuromyelitis Optica ^a^	0	0.00	0.00 (0.00–4158.15)
Optic Neuritis ^a^	2	0.18	11.38 (1.38–41.10)
Other Arterial Thromboembolism ^b^	4	9.19	0.44 (0.12–1.11)
Other Venous Thromboembolism ^a^	4	6.42	0.62 (0.17–1.59)
Polyneuropathies and Peripheral Neuropathies ^b^	14	8.89	1.57 (0.86–2.64)
Respiratory Failure ^b^	23	31.51	0.73 (0.46–1.10)
Rheumatoid Arthritis and Polyarthritis ^b^	17	29.22	0.58 (0.34–0.93)
Seizures ^b^	9	15.69	0.57 (0.26–1.09)
Stroke (Haemorrhagic) ^a^	25	23.94	1.04 (0.68–1.54)
Stroke (Ischaemic) ^b^	52	95.19	0.55 (0.41–0.72)
Subsequent Myocardial Infarction ^a^	0	1.15	0.00 (0.00–3.20)
Thrombocytopenia ^a^	8	11.99	0.67 (0.29–1.31)
Transient Ischaemic Attack ^b^	22	21.44	1.03 (0.64–1.55)
Transverse Myelitis ^a^	0	0.05	0.00 (0.00–76.21)
Vasculitis ^a^	2	1.04	1.93 (0.23–6.96)

^a^ Total background rate period: 1 January 2015 to 31 December 2023 inclusive. (Figure 1 scenario A). ^b^ COVID-19 vaccination background rate period: 1 January 2021 to 31 December 2023 inclusive. (Figure 1 scenario C). ^c^ Total background rate period excluding the COVID-19 pandemic period: 1 January 2015 to 31 December 2023, excluding 1 January 2020 to 31 December 2020. (Figure 1 scenario B). ^1.^ The risk period for all AESI was 1–28 days, except for seizures, which had a 0–6 day risk period. ^2^ Bold values indicate an identified safety signal.

**Table 3:** Statistically significant results from the SCCS analysis of AESI in post-RSV-vaccination risk periods among eligible 74–80-year-olds in Scotland, 01 August to 31 December 2024. (Unadjusted results).

Interval in Days	Events/Person Days	Incidence Rate Ratio (95% CI)	*p*
Acute Coronary Syndrome			
Baseline (−75 to −15)	474/100,711	Ref	-
**Clearance (−14 to −1)**	**77/23,114**	**0.71 (0.51–0.97)**	**0.0048**
0	1/1651	0.13 (0.01–1.69)	0.0402
**1–42**	**398/68,684**	**1.23 (1.03–1.47)**	**0.0023**
Acute Myocardial Infarction			
Baseline (−75 to −15)	149/35,990	Ref	-
**Clearance (−14 to −1)**	**10/8260**	**0.29 (0.13–0.68)**	**0.0002**
0	1/590	0.41 (0.03–5.43)	0.3732
1–7	17/4124	0.99 (0.51–1.92)	0.9820
**8–42**	**125/20,406**	**1.49 (1.09–2.03)**	**0.0011**
Baseline (−75 to −15)	149/35,990	Ref	-
**Clearance (−14 to −1)**	**10/8260**	**0.29 (0.13–0.68)**	**0.0002**
0	1/590	0.41 (0.03–5.43)	0.3732
**1–42**	**142/24,530**	**1.40 (1.04–1.90)**	**0.0039**
Acute Renal Failure			
Baseline (−75 to −15)	457/107,909	Ref	-
**Clearance (−14 to −1)**	**55/24,766**	**0.52 (0.36–0.76)**	**0.0000**
0	2/1769	0.27 (0.04–1.66)	0.0623
1–7	34/12,350	0.65 (0.41–1.03)	0.0157
**8–21**	**133/24,461**	**1.30 (1.01–1.67)**	**0.0085**
**22–42**	**189/36,117**	**1.27 (1.01–1.59)**	**0.0065**
**beyond risk periods**	**826/131,701**	**1.59 (1.36–1.86)**	**0.0000**
Baseline (−75 to −15)	457/107,909	Ref	-
**Clearance (−14 to −1)**	**55/24,766**	**0.52 (0.36–0.76)**	**0.0000**
0	2/1769	0.27 (0.04–1.66)	0.0623
1–21	167/36,811	1.08 (0.85–1.36)	0.4050
**22–42**	**189/36,117**	**1.27 (1.01–1.58)**	**0.0066**
**beyond risk periods**	**826/131,701**	**1.59 (1.36–1.86)**	**0.0000**
Baseline (−75 to −15)	457/107,909	Ref	-
**Clearance (−14 to −1)**	**55/24,766**	**0.52 (0.36–0.76)**	**0.0000**
0	2/1769	0.27 (0.04–1.66)	0.0623
1–42	356/72,928	1.17 (0.97–1.40)	0.0269
**beyond risk periods**	**826/131,701**	**1.59 (1.36–1.85)**	**0.0000**
Guillain–Barré Syndrome			
Baseline (−75 to −15)	1/976	Ref	-
Clearance (−14 to −1)	0/224	0.00 (0.00–Inf)	0.9984
0	0/16	0.00 (0.00–Inf)	0.9996
**1–42**	**10/634**	**16.60 (1.10–249.41)**	**0.0076**
43–90	1/672	1.76 (0.04–69.94)	0.6942
Baseline (−75 to −15)	1/976	Ref	-
Clearance (−14 to −1)	0/224	0.00 (0.00–Inf)	0.9985
0	0/16	0.00 (0.00–Inf)	0.9996
1–90	11/1306	10.62 (0.69–164.46)	0.0263
Heart Failure			
Baseline (−75 to −15)	367/76,128	Ref	-
**Clearance (−14 to −1)**	**58/17,472**	**0.69 (0.48–0.99)**	**0.0082**
0	0/1248	0.00 (0.00–Inf)	0.9835
**1–42**	**318/51,590**	**1.29 (1.06–1.57)**	**0.0010**
Stroke (Haemorrhagic)			
Baseline (−75 to −15)	26/9150	Ref	-
Clearance (−14 to −1)	3/2100	0.50 (0.10–2.42)	0.2594
0	0/150	0.00 (0.00–Inf)	0.9953
**1–7**	**11/1042**	**3.82 (1.51–9.67)**	**0.0002**
**8–42**	**35/4981**	**2.82 (1.42–5.60)**	**0.0001**
Baseline (−75 to −15)	26/9150	Ref	-
Clearance (−14 to −1)	3/2100	0.50 (0.10–2.42)	0.2594
0	0/150	0.00 (0.00–Inf)	0.9952
**1–42**	**46/6023**	**3.03 (1.58–5.79)**	**0.0000**

Bold values indicate statistically significant differences at *p* < 0.01.

**Table 4:** SCCS results with temporal adjustment for those AESI that signalled without temporal adjustments during post-RSV-vaccination risk periods among eligible 74–80-year-olds in Scotland, 1 August to 31 December 2024.

Interval in Days	Events/Person Days	Incidence Rate Ratio (95% CI)	*p*
Acute Coronary Syndrome			
Baseline (−75 to −15)	474/100,711	Ref	-
**Clearance (−14 to −1)**	**77/23,114**	0.68 (0.46–1.01)	0.0122
0	1/1651	0.12 (0.01–1.61)	0.0355
**1–42**	**398/68,684**	1.16 (0.75–1.81)	0.3748
Acute Myocardial Infarction			
Baseline (−75 to −15)	149/35,990	Ref	-
**Clearance (−14 to −1)**	**10/8260**	**0.29 (0.11–0.75)**	**0.0008**
0	1/590	0.39 (0.03–5.55)	0.3650
1–7	17/4124	0.95 (0.39–2.33)	0.8893
**8–42**	**125/20,406**	1.58 (0.68–3.65)	0.1603
Baseline (−75 to −15)	149/35,990	Ref	-
**Clearance (−14 to −1)**	**10/8260**	**0.28 (0.11–0.71)**	**0.0005**
0	1/590	0.36 (0.03–5.11)	0.3246
**1–42**	**142/24,530**	1.24 (0.57–2.70)	0.4721
Acute Renal Failure			
Baseline (−75 to −15)	457/107,909	Ref	-
**Clearance (−14 to −1)**	**55/24,766**	**0.49 (0.32–0.75)**	**0.0000**
0	2/1769	0.25 (0.04–1.60)	0.0551
1–7	34/12,350	0.63 (0.36–1.10)	0.0317
**8–21**	**133/24,461**	1.36 (0.88–2.12)	0.0717
**22–42**	**189/36,117**	1.54 (0.91–2.60)	0.0338
**beyond risk periods**	**826/131,701**	**2.16 (1.17–3.97)**	**0.0012**
Baseline (−75 to −15)	457/107,909	Ref	-
**Clearance (−14 to −1)**	**55/24,766**	**0.48 (0.31–0.74)**	**0.0000**
0	2/1769	0.24 (0.04–1.54)	0.0486
1–21	167/36,811	1.03 (0.68–1.55)	0.8682
**22–42**	**189/36,117**	1.35 (0.80–2.27)	0.1394
**beyond risk periods**	**826/131,701**	**1.85 (1.01–3.39)**	**0.0088**
Baseline (−75 to −15)	457/107,909	Ref	-
**Clearance (−14 to −1)**	**55/24,766**	**0.47 (0.30–0.72)**	**0.0000**
0	2/1769	0.23 (0.04–1.48)	0.0420
1–42	356/72,928	1.04 (0.69–1.57)	0.8146
**beyond risk periods**	**826/131,701**	1.48 (0.86–2.54)	0.0622
Guillain–Barré Syndrome			
Baseline (−75 to −15)	1/976	Ref	-
Clearance (−14 to −1)	0/224	0.00 (0.00–Inf)	0.9991
0	0/16	0.00 (0.00–Inf)	0.9999
**1–42**	**10/634**	**21.21 (1.15–389.63)**	**0.0069**
43–90	1/672	0.00 (0.00–Inf)	0.9992
Baseline (−75 to −15)	1/976	Ref	-
Clearance (−14 to −1)	0/224	0.00 (0.00–Inf)	0.9987
0	0/16	0.00 (0.00–Inf)	0.9999
**1–90**	**11/1306**	**23.95 (1.28–447.52)**	**0.0052**
Heart Failure			
Baseline (−75 to −15)	367/76,128	Ref	-
Clearance	58/17,472	0.72 (0.45–1.13)	0.0593
0	0/1248	0.00 (0.00–Inf)	0.9894
1–42	318/51,590	1.18 (0.70–1.98)	0.4052
Stroke (Haemorrhagic)			
Baseline (−75 to −15)	26/9150	Ref	-
Clearance (−14 to −1)	3/2100	0.61 (0.10–3.82)	0.4914
0	0/150	0.00 (0.00–Inf)	0.9954
**1–7**	**11/1042**	5.55 (1.00–30.85)	0.0100
**8–42**	**35/4981**	3.79 (0.57–25.39)	0.0713
Baseline (−75 to −15)	26/9150	Ref	-
Clearance (−14 to −1)	3/2100	0.66 (0.11–4.09)	0.5577
0	0/150	0.00 (0.00–Inf)	0.9954
**1–42**	**46/6023**	5.05 (0.89–28.71)	0.0164

Bold values indicate statistically significant differences at *p* < 0.01.

## Data Availability

The datasets presented in this article are not readily available because of information governance legislation. Access to NHS Scotland data is subject to approval by the NHS Scotland Public Benefit and Privacy Panel, by application through the Scotland National Safe Haven.

## Supplementary Materials

The following supporting information can be downloaded at: https://www.mdpi.com/article/10.3390/vaccines13111088/s1, Table S1: Pre-specified adverse events of special interest (AESI) and the International Classification of Diseases (ICD-10) Codes used for their identification, clean window and post-vaccination risk period(s); Table S2: Observed versus expected analysis—step-by-step methods, datasets and procedures planned to explore RSV vaccine safety in older adults in Scotland; Table S3: Self-controlled case series analysis—step-by-step methods, datasets and procedures planned to explore RSV vaccine safety in older adults in Scotland; Table S4: Results from self-controlled case series for all AESI with three or more events in their longest post-RSV-vaccination risk period among eligible 74 to 80-year-olds in Scotland, 1 August to 31 December 2024. (Unadjusted results).

## Abbreviations

The following abbreviations are used in this manuscript:

ADEM	Acute Disseminated Encephalomyelitis
AESI	Adverse Event of Special Interest
AMI	Acute Myocardial Infarction
CI	Confidence Interval
DVT	Deep Vein Thrombosis
GBS	Guillain Barré Syndrome
ICD	International Classification of Diseases
IR	Incidence Rates
IRR	Incident Rate Ratio
LRTI	Lower Respiratory Tract Infection
MHRA	Medicines and Healthcare products Regulatory Agency
NCDS	National Clinical Data Store
NRS	National Records of Scotland
OE	Observed Versus Expected
PROMISE	Preparing for RSV Immunisation and Surveillance in Europe
RSV	Respiratory Syncytial Virus
RSVpreF	Respiratory Syncytial Virus Bivalent Prefusion F Vaccine
SCCS	Self-controlled Case Series
SMR01	Scottish Morbidity Record 01
US	United States
VMT	Vaccine Management Tool
