# Supplementary material for: RSV Vaccination Programme for Older Adults: A Scotland-Wide Study on RSVpreF Vaccine Safety
Source: Vaccines (Basel). 2025 Oct 24;13(11):1088. doi: 10.3390/vaccines13111088 (PMC12656231; doi:10.3390/vaccines13111088)
Supplement: Supplementary file 1 [file vaccines-13-01088-s001.zip › vaccines-3908897-supplementary.pdf]

Supplementary Table S1: Pre-specified Adverse Events of Special Interest (AESI) and the International Classification of Diseases (ICD-10) Codes used for their identification, clean window and post-vaccination risk period(s).

#	AESI	Outcome	ICD-10 Codes	Clean Window	SCCS post-vaccination risk period(s) in days
**1**	**Guillain-Barre Syndrome (GBS)**	Acute (Post-) infective polyneuritis	G61.0	365 days	1-42; 43-90
		Disorders of multiple cranial nerves| Polyneuritis cranialis	G52.7		
**2**	**Thrombocytopenia**	Thrombotic microangiopathy	M31.1	365 days	1-21; 22-42
		Idiopathic thrombocytopenic purpura	D69.3		
		Other primary thrombocytopenia	D69.4		
		Secondary thrombocytopenia	D69.5		
		Thrombocytopenia unspecified	D69.6		
**3**	**Facial nerve disorders including Bell's Palsy**	Bell palsy	G51.0	183 days	1-7; 8-42; 43-90
		Injury of facial nerve/7th cranial nerve	**G51**		
**4**	**Deep Vein Thrombosis and Pulmonary Embolism**	Pulmonary embolism with mention of acute cor pulmonale	I26.0	365 days	1-42
		Pulmonary embolism without mention of acute cor pulmonale	I26.9		
		Phlebitis and thrombophlebitis of femoral vein	I80.1		
		Phlebitis and thrombophlebitis of other deep vessels of lower extremities	I80.2		
		Phlebitis and thrombophlebitis of lower extremities, unspecified	I80.3		
**5**	**Other Venous Thromboembolism**	Phlebitis and thrombophlebitis of other sites	I80.8	365 days	1-42
		Phlebitis and thrombophlebitis of unspecified site	I80.9		
		Portal vein thrombosis	I81		

		Budd Chiari syndrome	I82.0		
		Thrombophlebitis migrans	I82.1		
		Embolism and thrombosis of vena cava	I82.2		
		Embolism and thrombosis of renal vein	I82.3		
		Embolism and thrombosis of other specified veins	I82.8		
		Embolism and thrombosis of unspecified vein	I82.9		
		Deep phlebothrombosis in pregnancy	D22.3		
6	**Intracranial Venous Thrombosis**	Cerebral infarction due to cerebral venous thrombosis, nonpyogenic	I63.6	365 days	1-42
		Intracranial and intraspinal phlebitis and thrombophlebitis	G08		
		Nonpyogenic thrombosis of intracranial venous system	I67.6		
**7**	**Disseminated Intravascular Coagulation**	Disseminated intravascular coagulation (defribrination syndrome)	D65	365 days	1-21; 22-42
**8**	**Narcolepsy**	Narcolepsy and cataplexy	G47.4	365 days	1-42
**9**	**Encephalitis including Acute Disseminated Encephalomyelitis (ADEM)**	Acute disseminated encephalitis	G040	183 days	1-28
		Other encephalitis, myelitis and encephalomyelitis	G048		
		Encephalitis, myelitis and encephalomyelitis, unspecified	G049		
		Acute and subacute haemorrhagic leukoencephalitis	G36.1		
**10**	**Angina Pectoris**	Angina Pectoris	**I20**	365 days	1-28
		Unstable angina	I20.0		
		Angina pectoris with documented spasm	I20.1		
		Other forms of angina pectoris	I20.8		
		Angina pectoris, unspecified	I20.9		
**11**	**Acute Myocardial Infarction**	Acute myocardial infarction	**I21**	365 days	1-7; 8-42
		Acute transmural myocardial infarction of anterior wall	I21.0		
		Acute transmural myocardial infarction of inferior wall	I21.1		
		Acute transmural myocardial infarction of other sites	I21.2		

		Acute transmural myocardial infarction of unspecified site	I21.3		
		Acute sub endocardial myocardial infarction	I21.4		
		Acute myocardial infarction, unspecified	I21.9		
**12**	**Subsequent Myocardial Infarction**	Subsequent myocardial infarction	**I22**	365 days	1-7; 8-42
		Subsequent myocardial infarction of anterior wall	I22.0		
		Subsequent myocardial infarction of inferior wall	I22.1		
		Subsequent myocardial infarction of other sites	I22.8		
		Subsequent myocardial infarction of unspecified site	I22.9		
**13**	**Acute Coronary Syndrome**	Haemopericardium as current complication following acute myocardial infarction	I23.0	365 days	1-42
		Atrial septal defect as current complication following acute myocardial infarction	I23.1		
		Ventricular septal defect as current complication following acute myocardial infarction	I23.2		
		Rupture of cardiac wall without haemopericardium as current complication following acute myocardial infarction	I23.3		
		Rupture of chordae tendineae as current complication following acute myocardial infarction	I23.4		
		Rupture of papillary muscle as current complication following acute myocardial infarction	I23.5		
		Thrombosis of atrium, auricular appendage, and ventricle as current complications following acute myocardial infarction	I23.6		
		Other current complications following acute myocardial infarction	I23.8		

		Coronary thrombosis not resulting in myocardial infarction	I24.0		
		Dressler syndrome	I24.1		
		Other forms of acute ischaemic heart disease/Acute coronary syndrome	I24.8		
		Acute ischaemic heart disease, unspecified | syndrome | coronary | acute NEC	I24.9		
		Acute myocardial infarction	I21		
		Angina Pectoris	I20		
**14**	**Myocarditis and Pericarditis**	Acute pericarditis	**I30**	365 days	1-42
		Acute nonspecific idiopathic pericarditis	I30.0		
		Other forms of acute pericarditis	I30.8		
		Acute pericarditis, unspecified	I30.9		
		Dressler syndrome	I24.1		
		Cardiomyopathy due to drugs and other external agents	I42.7		
		Acute myocarditis	**I40**		
		Isolated myocarditis	I40.1		
		Other acute myocarditis	I40.8		
		Acute myocarditis, unspecified	I40.9		
		Myocarditis, unspecified	I51.4		
**15**	**Heart Failure**	Heart failure	**150**	365 days	1-42
		Congestive heart failure	I50.0		
		Left ventricular failure	I50.1		
		Heart failure, unspecified	I50.9		
**16**	**Transient Ischaemic Attack**	Transient cerebral ischaemic attacks and related syndromes	**G45**	365 days	1-42
		Vertebro-basilar artery syndrome	G45.0		
		Carotid artery syndrome (hemispheric)	G45.1		
		Multiple and bilateral precerebral artery syndromes	G45.2		
		Amaurosis fugax	G45.3		
		Transient global amnesia	G45.4		

		Other transient cerebral ischaemic attacks and related syndromes	G45.8		
		Transient cerebral ischaemic attack, unspecified	G45.9		
**17**	**Stroke (haemorrhagic)**	**Subarachnoid haemorrhage**	**I60**	365 days	1-7; 8-42
		Subarachnoid haemorrhage from carotid siphon and bifurcation	I60.0		
		Subarachnoid haemorrhage from middle cerebral artery	I60.1		
		Subarachnoid haemorrhage from anterior communicating artery	I60.2		
		Subarachnoid haemorrhage from posterior communicating artery	I60.3		
		Subarachnoid haemorrhage from basilar artery	I60.4		
		Subarachnoid haemorrhage from vertebral artery	I60.5		
		Subarachnoid haemorrhage from other intracranial arteries	I60.6		
		Subarachnoid haemorrhage from intracranial artery, unspecified	I60.7		
		Other subarachnoid haemorrhage	I60.8		
		Subarachnoid haemorrhage, unspecified	I60.9		
		**Intracerebral haemorrhage**	**I61**		
		Intracerebral haemorrhage in hemisphere, subcortical	I61.0		
		Intracerebral haemorrhage in hemisphere, cortical	I61.1		
		Intracerebral haemorrhage in hemisphere, unspecified	I61.2		
		Intracerebral haemorrhage in brain stem	I61.3		
		Intracerebral haemorrhage in cerebellum	I61.4		
		Intracerebral haemorrhage, intraventricular	I61.5		
		Intracerebral haemorrhage, multiple localized	I61.6		

		Other intracerebral haemorrhage	I61.8		
		Intracerebral haemorrhage, unspecified	I61.9		
		**Intracranial haemorrhage**			
		Other non-traumatic intracranial haemorrhage	**I62**		
		Non traumatic subdural haemorrhage	I62.0		
		Non traumatic extradural haemorrhage	I62.1		
		Intracranial haemorrhage	I62.9		
**18**	**Multiple Sclerosis**	Multiple Sclerosis	G35	365 days	1-42; 43-90
		Acute disseminated demyelination, unspecified	G36.9		
**19**	**Neuromyelitis Optica**	Neuromyelitis optica [Devic]	G36.0	365 days	1-42
**20**	**Other Arterial Thromboembolism**	Transient retinal artery occlusion	H34.0	365 days	1-42
		Central retinal artery occlusion	H34.1		
		Other retinal artery occlusions	H34.2		
		Retinal vascular occlusion, unspecified	H34.9		
		Embolism and thrombosis of abdominal aorta	I74.0		
		Embolism and thrombosis of other and unspecified parts of aorta	I74.1		
		Embolism and thrombosis of arteries of upper extremities	I74.2		
		Embolism and thrombosis of arteries of lower extremities	I74.3		
		Embolism and thrombosis of arteries of extremities, unspecified	I74.4		
		Embolism and thrombosis of iliac artery	I74.5		
		Embolism and thrombosis of other arteries	I74.8		
		Embolism and thrombosis of unspecified artery	I74.9		
		Ischaemia and infarction of kidney	N28.0		
**21**	**Optic Neuritis**	Optic neuritis NOS	H46	365 days	1-42; 43-90

**22**	**Transverse Myelitis**	Acute transverse myelitis in demyelinating disease of central nervous system	G37.3	365 days	1-42; 43-90
**23**	**Polyneuropathies and Peripheral Neuropathies**	Polyneuropathies and other disorders of the peripheral nervous system	G60-64	365 days	1-42
		Hereditary and idiopathic neuropathy	**G60**		
		Inflammatory polyneuropathy	**G61**		
		Serum neuropathy	G61.1		
		Other inflammatory polyneuropathies	G61.8		
		Inflammatory polyneuropathy, unspecified	G61.9		
		Other neuropathies	**G62**		
		Polyneuropathy in diseases classified elsewhere	**G63**		
		Other disorders of peripheral nervous system	**G64**		
		Idiopathic peripheral autonomic neuropathy	G90.0		
		Disorder of autonomic nervous system, unspecified	G90.9		
**24**	**Rheumatoid Arthritis and Polyarthritis**	**Seropositive rheumatoid arthritis**	**M05**	365 days	1-42; 43-90
		Felty syndrome	M05.0		
		Rheumatoid lung disease	M05.1		
		Rheumatoid vasculitis	M05.2		
		Rheumatoid arthritis with involvement of other organs and systems	M05.3		
		Other seropositive rheumatoid arthritis	M05.8		
		Seropositive rheumatoid arthritis, unspecified	M05.9		
		Other rheumatoid arthritis	**M06**		
		Seronegative rheumatoid arthritis	M06.0		
		Adult-onset Still disease	M06.1		
		Rheumatoid bursitis	M06.2		
		Rheumatoid nodule	M06.3		
		Inflammatory polyarthropathy	M06.4		
		Other specified rheumatoid arthritis	M06.8		

		Rheumatoid arthritis, unspecified	M06.9		
**25**	**Myasthenia Gravis**	Myasthenia gravis and other myoneural disorders	**G70**	365 days	1-42
		Myasthenia gravis	G70.0		
		Myoneural disorder, unspecified	G70.9		
		Myasthenic syndromes in other diseases classified elsewhere	G73.3		
**26**	**Autoimmune Thyroiditis**	autoimmune thyroiditis	E06.3	365 days	1-42; 43-90
		thyroiditis, unspecified	E06.9		
		Postpartum thyroiditis	O90.5		
**27**	**Seizures**	Epilepsy	**G40**	28 days	0-6
		Localization-related (focal)(partial) idiopathic epilepsy and epileptic syndromes with seizures of localized onset	G40.0		
		Localization-related (focal)(partial) symptomatic epilepsy and epileptic syndromes with simple partial seizures	G40.1		
		Localization-related (focal)(partial) symptomatic epilepsy and epileptic syndromes with complex partial seizures	G40.2		
		Generalized idiopathic epilepsy and epileptic syndromes	G40.3		
		Other generalized epilepsy and epileptic syndromes	G40.4		
		Special epileptic syndromes	G40.5		
		Grand mal seizures, unspecified (with or without petit mal)	G40.6		
		Petit mal, unspecified, without grand mal seizures	G40.7		
		Other epilepsy	G40.8		
		Epilepsy, unspecified	G40.9		
		Status epilepticus	**G41**		
		Grand mal status epilepticus	G41.0		
		Petit mal status epilepticus	G41.1		

		Complex partial status epilepticus	G41.2		
		Other status epilepticus	G41.8		
		Status epilepticus, unspecified	G41.9		
		Convulsions, not elsewhere classified	**R56**		
**28**	**Fibromyalgia**	Fibromyalgia	M79.7	365 days	1-91; 92-181
		Myalgia	M79.1		
		Myositis	M60		
**29**	**Chronic Fatigue Syndrome**	Post viral fatigue syndrome	G93.3	365 days	1-91; 92-181
**30**	**Demyelination**	Other specified demyelinating diseases of central nervous system	G37.8	365 days	1-42;43-90
		Demyelinating disease of central nervous system, unspecified	G37.9		
**31**	**Vasculitis**	Skin vasculitis	L95.9, L95.8, **L95**	365 days	1-7; 8-28
		Allergic vasculitis	M31.0		
		Henoch-Schoenlein Purpura	D69.0		
**32**	**Stroke (Ischemic)**	**Cerebral infarction**		365 days	1-7; 8-42
		Cerebral infarction due to thrombosis of pre-cerebral arteries	I63.0		
		Cerebral infarction due to embolism of pre cerebral arteries	I63.1		
		Cerebral infarction due to unspecified occlusion or stenosis of pre cerebral arteries	I63.2		
		Cerebral infarction due to thrombosis of cerebral arteries	I63.3		
		Cerebral infarction due to embolism of cerebral arteries	I63.4		
		Cerebral infarction due to unspecified occlusion or stenosis of cerebral arteries	I63.5		
		Other cerebral infarction	I63.8		

		Cerebral infarction, unspecified	I63.9		
		Stroke, not specified as haemorrhage or infarction	I64		
		Occlusion and stenosis of vertebral artery	I65.0		
		Occlusion and stenosis of basilar artery	I65.1		
		Occlusion and stenosis of carotid artery	I65.2		
		Occlusion and stenosis of multiple and bilateral precerebral arteries	I65.3		
		Occlusion and stenosis of other precerebral artery	I65.8		
		Occlusion and stenosis of unspecified precerebral artery	I65.9		
		Occlusion and stenosis of middle cerebral artery	I66.0		
		Occlusion and stenosis of anterior cerebral artery	I66.1		
		Occlusion and stenosis of posterior cerebral artery	I66.2		
		Occlusion and stenosis of cerebellar arteries	I66.3		
		Occlusion and stenosis of multiple and bilateral cerebral arteries	I66.4		
		Occlusion and stenosis of other cerebral artery	I66.8		
		Occlusion and stenosis of unspecified cerebral artery	I66.9		
**33**	**Respiratory Failure**	Respiratory failure unspecified	J96.9	365 days	1-21; 22-42
		Adult Respiratory Distress Syndrome	J80		
**34**	**Acute and Subacute Hepatic Failure**	Acute and subacute hepatic failure	K72.0	365 days	1-7; 8-21; 22-42
		Postpartum acute renal failure	O90.4		
**35**	**Acute Renal Failure**	**Acute renal failure**	**N17**	365 days	1-7; 8-21; 22-42
		Acute renal failure with tubular necrosis	N17.0		
		Acute renal failure with acute cortical necrosis	N17.1		

		Acute renal failure with medullary necrosis	N17.2		
		Other acute renal failure	N17.8		
		Acute renal failure, unspecified	N17.9		
		Unspecified kidney failure	**N19**		
**36**	**Lymphadenopathy**	Enlarged lymph nodes	R59	365 days	1-7, 8-21, 22-42
		LOCALIZED ENLARGED LYMPH NODES	R59.0		
		GENERALIZED ENLARGED LYMPH NODES	R59.1		
		ENLARGED LYMPH NODES, UNSPECIFIED	R59.9		
**37**	**Anaphylactic shock**	Anaphylactic shock, unspecified	**T78.2**	30 days	0-1
		Anaphylactic shock due to adverse effect of correct drug or medicament properly administered	T88.6		
**38**	**Atrial Fibrillation**	Paroxysmal atrial fibrillation	I48.0	365 days	1-7; 8-21; 22-42
		Persistent atrial fibrillation	I48.1		
		Atrial fibrillation and atrial flutter (unspecified)	I48.9		
		Atrial fibrillation and flutter	I48.X		
**39**	**Bulbar Palsy**	Other inherited spinal muscular atrophy<br>Progressive bulbar palsy of childhood [Fazio-Londe]<br>Spinal muscular atrophy:<br>adult form<br>childhood form, type II<br>distal<br>juvenile form, type III [Kugelberg-Welander]<br>scapuloperoneal form	G12.1	183 days	1-7; 8-42; 43-90
		Motor neuron disease<br>Familial motor neuron disease<br>Lateral sclerosis:<br>amyotrophic<br>primary<br>Progressive:<br>bulbar palsy<br>spinal muscular atrophy	G12.2		

Supplementary Table S2: Observed versus expected analysis - step-by-step methods, datasets and procedures planned to explore RSV vaccine safety in older adults in Scotland.

1. Identification of patients aged 75 to 79 years-old with a hospital stay containing a diagnosis for an AESI using the Scottish Morbidity Record 01 (SMR01) national dataset.	The SMR01 dataset includes data on discharge diagnoses for all inpatient and day patient episodes from acute specialties from hospitals in Scotland, excluding obstetric and psychiatric specialties. Episode level hospital data will be extracted from SMR01 from 01 July 2013 to capture hospital stays in their entirety and include a one-year lookback period to apply the clean window to all hospital admissions (described in Step 2) prior to the background rate periods. An episode is a period of hospital care initiated by a referral (including re-referral) or admission and ended by a discharge.[13]<br><br>AESI events are identified based on the presence of any relevant ICD-10 diagnostic code within any episode. SMR01 data will be aggregated from episodes to hospital stay level for each individual based on their unique Community Health Index (CHI) number, counting multiple events within a hospital stay once for each AESI. Age is defined as the age at the date of hospital admission.
2. Application of a clean window to all hospital stays for each AESI.	For each individual AESI, the time between hospital admissions is calculated. Only those admissions that occur at least a clean window later than the previous admission is included as an event in the incidence rates for each AESI. This limits the incidence rates being inflated by repeat admissions for exacerbations of the same AESI occurrence.
3. Calculation of mean background incidence rates (IRs) by age group (75-79 years) and sex, by month.	The mean monthly incidence rate of hospital admissions per 100,000 PYRS (person-years) for each AESI is calculated by age and sex for background rate time periods of interest. One of the following background rate periods will be chosen following the criteria outlined in Figure 1:<br>a) 01 January 2015 to 31 December 2023<br>b) 01 January 2015 to 31 December 2023, excluding 01 January 2020 to 31 December 2020.<br>c) 01 January 2021 to 31 December 2023.<br>The denominator is the NRS mid-year population estimate for ages 75 to79 years-old for each respective year. Where estimates are not yet published for recent year(s), the latest population estimate is used.
5. Calculation of observed events: Identification of vaccinations delivered	Data on RSVpreF vaccinations administered to 75- to 79-year-olds in Scotland will be extracted from the National Clinical Data Store from 01 August 2024 onwards. These data are recorded using the VMT, a web-based tool for healthcare staff in Scotland

to 75 to 79-year-olds and linkage with AESI related hospital stays.	to record real-time patient vaccination data at the point of care[11].<br><br>AESI-related hospital stays with an admission date from 01 August 2024 onwards will be linked to RSV vaccination records using the CHI number to calculate the observed number of AESI cases.
6. Methods for Observed Expected Analysis	The vaccinated population is multiplied by the IRs for each background rate period in turn to calculate the expected rate of events for each AESI in the post-vaccination risk period (0-6 days for seizures and 1-28 days for all other AESI). These are compared to the observed number of events in the post-vaccination risk period using the observed expected (OE) ratio, by dividing the observed value by the expected value. OE ratios will be calculated with 95% confidence intervals. Lower confidence interval (CI) bounds > 1.0 suggest an increase in the AESI events above the expected rate. Lower CI > 1.0 and three or more events in the post-vaccination risk period define a signal for further investigation.
7. Statistical packages used	All analyses will be conducted in R within the Posit workbench environment using the tidyverse, survival and lubridate packages.

Supplementary Table S3: Self-controlled case series analysis - step-by-step methods, datasets and procedures planned to explore RSV vaccine safety in older adults in Scotland.

1. Identification of patients aged 74-80 years with a hospital stay containing a diagnosis for an AESI using the Scottish Morbidity Record 01 (SMR01) national dataset	The SMR01 dataset includes data on discharge diagnoses for all inpatient and day patient episodes from acute specialties from hospitals in Scotland, excluding obstetric and psychiatric specialties. Episode level hospital data will be extracted from SMR01 for the period 01 November 2023 onwards to capture hospital stays in their entirety prior to the baseline period starting on 18 May 2024 (75 days prior to the programme start on 01 August). An episode is a period of hospital care initiated by a referral (including re-referral) or admission and ended by a discharge.[13]<br><br>AESI events are identified based on the presence of a relevant ICD-10 code within any episode. SMR01 data will be aggregated from episodes to hospital stay level for each individual based on their unique CHI number, counting multiple events within a hospital stay once for each AESI. Age is defined as the age at the date of hospital admission.
2. Identification of vaccinations delivered to 74 to 80-year-olds and linkage with AESI related hospital stays	Data on RSVpreF vaccinations administered to 74- to 80-year-olds in Scotland will be extracted from the NCDS from 01 August 2024 onwards. (Note that individuals are eligible for RSV vaccination if they turned 75 on or after the 1 August 2024, up to and including 31 July 2025. Therefore some 74-year-olds will be eligible during the programme. Similarly, individuals aged 79 years old on 01 August 2024 will remain eligible even after they turn 80 years old). These data are recorded using the VMT, a web-based tool for healthcare staff in Scotland to record real-time patient vaccination data at the point of care[11]. AESI-related hospital stays with an admission date from 18 May 2024 (75 days prior to the vaccine programme start on 01 August 2024)

	onwards will be linked to RSVpreF vaccination records using the CHI number.
3. Methods for SCSS	Conditional logistic regression models will compare rates of hospital admissions in the post-vaccination risk periods with the baseline period, stratified by individual and calendar time (either 28 or 56 days). Incidence rate ratios (IRRs) will be estimated to quantify the rate of hospital stays in the risk period relative to the baseline period. Individuals will be censored on the earliest of the following: date of death, study end, date of leaving Scotland. A p-value of <0.01 will be used as the threshold of significance, indicating there is a significant increase in post-vaccination hospitalisations for the AESI and thus indicating a signal for further investigation.
4. Statistical packages used	All analyses will be conducted in R within the Posit workbench environment using the tidyverse, survival and lubridate packages.

Supplementary Table S4: Results from self-controlled case series analysis for all AESI with three or more events in their longest post-RSV-vaccination risk period among eligible 74 to 80-year-olds in Scotland, 1 August to 31 December 2024. (Unadjusted results).

Interval in Days	Events/Person days	Incidence Rate Ratio (99% CI)	*p*
	Acute Coronary Syndrome		
Baseline (−75 to −15)	474/100,711	Ref	-
**Clearance (−14 to −1)**	**77/23,114**	**0.71 (0.51-0.97)**	**0.0048**
0	1/1,651	0.13 (0.01-1.69)	0.0402
**1-42**	**398/68,684**	**1.23 (1.03-1.47)**	**0.0023**
	Acute Myocardial Infarction		
Baseline (−75 to −15)	149/35,990	Ref	-
**Clearance (−14 to −1)**	**10/8,260**	**0.29 (0.13-0.68)**	**0.0002**
0	1/590	0.41 (0.03-5.43)	0.3732
1-7	17/4,124	0.99 (0.51-1.92)	0.9820
**8-42**	**125/20,406**	**1.49 (1.09-2.03)**	**0.0011**
Baseline (−75 to −15)	149/35,990	Ref	-
**Clearance (−14 to −1)**	**10/8,260**	**0.29 (0.13-0.68)**	**0.0002**
0	1/590	0.41 (0.03-5.43)	0.3732
**1-42**	**142/24,530**	**1.40 (1.04-1.90)**	**0.0039**
	Acute Renal Failure		
Baseline (−75 to −15)	457/107,909	Ref	-
**Clearance (−14 to −1)**	**55/24,766**	**0.52 (0.36-0.76)**	**0.0000**
0	2/1,769	0.27 (0.04-1.66)	0.0623
1-7	34/12,350	0.65 (0.41-1.03)	0.0157
**8-21**	**133/24,461**	**1.30 (1.01-1.67)**	**0.0085**
**22-42**	**189/36,117**	**1.27 (1.01-1.59)**	**0.0065**
Baseline (−75 to −15)	457/107,909	Ref	-
**Clearance (−14 to −1)**	**55/24,766**	**0.52 (0.36-0.76)**	**0.0000**
0	2/1,769	0.27 (0.04-1.66)	0.0623
1-21	167/36,811	1.08 (0.85-1.36)	0.4050
**22-42**	**189/36,117**	**1.27 (1.01-1.58)**	**0.0066**
Baseline (−75 to −15)	457/107,909	Ref	-
**Clearance (−14 to −1)**	**55/24,766**	**0.52 (0.36-0.76)**	**0.0000**

Interval in Days	Events/Person days	Incidence Rate Ratio (99% CI)	*p*
0	2/1,769	0.27 (0.04-1.66)	0.0623
1-42	356/72,928	1.17 (0.97-1.40)	0.0269
Angina Pectoris			
Baseline (−75 to −15)	324/64,416	Ref	-
Clearance (−14 to −1)	64/14,784	0.86 (0.60-1.22)	0.2714
0	1/1,056	0.19 (0.01-2.48)	0.0947
1-28	151/29,391	1.02 (0.79-1.31)	0.8468
Atrial Fibrillation			
Baseline (−75 to −15)	826/163,846	Ref	-
**Clearance (−14 to −1)**	**124/37,604**	**0.65 (0.51-0.84)**	**0.0000**
0	0/2,686	0.00 (0.00-Inf)	0.9751
1-7	85/18,766	0.90 (0.67-1.20)	0.3467
8-21	216/37,297	1.15 (0.95-1.41)	0.0614
22-42	317/55,085	1.15 (0.97-1.37)	0.0308
Baseline (−75 to −15)	826/163,846	Ref	-
**Clearance (−14 to −1)**	**124/37,604**	**0.65 (0.51-0.84)**	**0.0000**
0	0/2,686	0.00 (0.00-Inf)	0.9750
1-21	301/56,063	1.07 (0.90-1.27)	0.3289
22-42	317/55,085	1.15 (0.97-1.37)	0.0310
Baseline (−75 to −15)	826/163,846	Ref	-
**Clearance (−14 to −1)**	**124/37,604**	**0.65 (0.51-0.84)**	**0.0000**
0	0/2,686	0.00 (0.00-Inf)	0.9750
1-42	618/111,148	1.11 (0.97-1.27)	0.0499
Bulbar Palsy			
Baseline (−75 to −15)	4/1,159	Ref	-
Clearance (−14 to −1)	0/266	0.00 (0.00-Inf)	0.9967
0	0/19	0.00 (0.00-Inf)	0.9991
1-7	1/133	2.18 (0.12-39.06)	0.4857
8-42	7/665	3.06 (0.61-15.42)	0.0747
43-90	6/849	2.26 (0.42-12.15)	0.2125
Baseline (−75 to −15)	4/1,159	Ref	-

Interval in Days	Events/Person days	Incidence Rate Ratio (99% CI)	*p*
Clearance **(−14 to −1)**	0/266	0.00 (0.00-Inf)	0.9967
0	0/19	0.00 (0.00-Inf)	0.9991
1-42	8/798	2.91 (0.60-14.14)	0.0811
43-90	6/849	2.26 (0.42-12.15)	0.2125
Baseline (−75 to −15)	4/1,159	Ref	-
Clearance (−14 to −1)	0/266	0.00 (0.00-Inf)	0.9979
0	0/19	0.00 (0.00-Inf)	0.9994
1-90	14/1,647	2.60 (0.60-11.29)	0.0933
Deep Vein Thrombosis (DVT) and Pulmonary Embolism			
Baseline (−75 to −15)	75/17,202	Ref	-
Clearance (−14 to −1)	10/3,948	0.58 (0.24-1.38)	0.1066
0	1/282	0.81 (0.06-10.88)	0.8373
1-42	59/11,696	1.16 (0.74-1.82)	0.3957
Facial nerve disorders including Bell's Palsy			
Baseline (−75 to −15)	5/1,769	Ref	-
Clearance (−14 to −1)	5/406	4.36 (0.85-22.28)	0.0199
0	0/29	0.00 (0.00-Inf)	0.9978
1-7	1/203	1.74 (0.10-29.33)	0.6120
8-42	4/1,015	1.39 (0.25-7.85)	0.6202
43-90	6/1,386	1.53 (0.32-7.31)	0.4794
Baseline (−75 to −15)	5/1,769	Ref	-
Clearance (−14 to −1)	5/406	4.36 (0.85-22.28)	0.0199
0	0/29	0.00 (0.00-Inf)	0.9978
1-42	5/1,218	1.45 (0.28-7.41)	0.5550
43-90	6/1,386	1.53 (0.32-7.31)	0.4794
Baseline (−75 to −15)	5/1,769	Ref	-
Clearance (−14 to −1)	5/406	4.36 (0.85-22.28)	0.0199
0	0/29	0.00 (0.00-Inf)	0.9978
1-90	11/2,604	1.50 (0.37-6.01)	0.4550
Fibromyalgia			
Baseline (−75 to −15)	24/4,270	Ref	-

Interval in Days	Events/Person days	Incidence Rate Ratio (99% CI)	*p*
Clearance (−14 to −1)	0/980	0.00 (0.00-Inf)	0.9961
0	0/70	0.00 (0.00-Inf)	0.9990
1-91	32/6,205	0.93 (0.46-1.87)	0.7803
92-181	12/2,059	1.06 (0.42-2.68)	0.8748
		Guillain-Barre Syndrome	
Baseline (−75 to −15)	1/976	Ref	-
Clearance (−14 to −1)	0/224	0.00 (0.00-Inf)	0.9984
0	0/16	0.00 (0.00-Inf)	0.9996
**1-42**	**10/634**	**16.60 (1.10-249.41)**	**0.0076**
43-90	1/672	1.76 (0.04-69.94)	0.6942
Baseline (−75 to −15)	1/976	Ref	-
Clearance (−14 to −1)	0/224	0.00 (0.00-Inf)	0.9985
0	0/16	0.00 (0.00-Inf)	0.9996
1-90	11/1,306	10.62 (0.69-164.46)	0.0263
		Heart Failure	
Baseline (−75 to −15)	367/76,128	Ref	-
**Clearance (−14 to −1)**	**58/17,472**	**0.69 (0.48-0.99)**	**0.0082**
0	0/1,248	0.00 (0.00-Inf)	0.9835
**1-42**	**318/51,590**	**1.29 (1.06-1.57)**	**0.0010**
		Lymphadenopathy	
Baseline (−75 to −15)	22/5,978	Ref	-
Clearance (−14 to −1)	5/1,372	0.99 (0.28-3.55)	0.9842
0	1/98	2.78 (0.20-38.87)	0.3183
1-7	2/686	0.79 (0.12-5.31)	0.7523
8-21	6/1,350	1.25 (0.38-4.11)	0.6297
22-42	8/1,974	1.16 (0.40-3.40)	0.7181
Baseline (−75 to −15)	22/5,978	Ref	-
Clearance (−14 to −1)	5/1,372	0.99 (0.28-3.55)	0.9842
0	1/98	2.78 (0.20-38.87)	0.3183
1-21	8/2,036	1.09 (0.38-3.17)	0.8327
22-42	8/1,974	1.16 (0.40-3.40)	0.7202

Interval in Days	Events/Person days	Incidence Rate Ratio (99% CI)	*p*
Baseline (−75 to −15)	22/5,978	Ref	-
Clearance (−14 to −1)	5/1,372	0.99 (0.28-3.55)	0.9842
0	1/98	2.78 (0.20-38.87)	0.3183
1-42	16/4,010	1.12 (0.48-2.64)	0.7223
Multiple Sclerosis			
Baseline (−75 to −15)	33/5,978	Ref	-
Clearance (−14 to −1)	2/1,372	0.26 (0.04-1.72)	0.0670
0	0/98	0.00 (0.00-Inf)	0.9950
1-42	29/4,114	1.28 (0.66-2.47)	0.3322
43-90	33/4,636	1.34 (0.71-2.55)	0.2347
Baseline (−75 to −15)	33/5,978	Ref	-
Clearance (−14 to −1)	2/1,372	0.26 (0.04-1.72)	0.0670
0	0/98	0.00 (0.00-Inf)	0.9950
1-90	62/8,750	1.31 (0.75-2.29)	0.2086
Myasthenia Gravis			
Baseline (−75 to −15)	8/1,586	Ref	-
Clearance (−14 to −1)	1/364	0.54 (0.04-8.38)	0.5659
0	0/26	0.00 (0.00-Inf)	0.9974
1-42	13/1,092	2.38 (0.74-7.59)	0.0550
Myocarditis and Pericarditis			
Baseline (−75 to −15)	6/1,586	Ref	-
Clearance (−14 to −1)	1/364	0.73 (0.04-11.75)	0.7668
0	0/26	0.00 (0.00-Inf)	0.9979
1-42	6/1,092	1.45 (0.33-6.45)	0.5174
Optic Neuritis			
Baseline (−75 to −15)	0/183	Ref	-
Clearance (−14 to −1)	0/42	1.00 (0.00-Inf)	1.0000
0	0/3	1.00 (0.00-Inf)	1.0000
1-42	3/119	2047025769.18 (0.00-Inf)	0.9992
43-90	0/50	0.94 (0.00-Inf)	1.0000
Baseline (−75 to −15)	0/183	Ref	-

Interval in Days	Events/Person days	Incidence Rate Ratio (99% CI)	*p*
Clearance (−14 to −1)	0/42	1.00 (0.00-Inf)	1.0000
0	0/3	1.00 (0.00-Inf)	1.0000
1-90	3/169	948586038.39 (0.00-Inf)	0.9990
Other Arterial Thromboembolism			
Baseline (−75 to −15)	15/3,599	Ref	-
Clearance (−14 to −1)	3/826	0.87 (0.17-4.44)	0.8277
0	0/59	0.00 (0.00-Inf)	0.9969
1-42	7/2,468	0.68 (0.21-2.23)	0.4082
Other Venous Thromboembolism			
Baseline (−75 to −15)	13/3,050	Ref	-
Clearance (−14 to −1)	1/700	0.33 (0.02-4.85)	0.2920
0	0/50	0.00 (0.00-Inf)	0.9971
1-42	8/2,052	0.92 (0.29-2.95)	0.8604
Polyneuropathies and Peripheral Neuropathies			
Baseline (−75 to −15)	42/5,368	Ref	-
Clearance (−14 to −1)	7/1232	0.72 (0.25-2.08)	0.4292
0	0/88	0.00 (0.00-Inf)	0.9964
1-42	37/3654	1.31 (0.73-2.35)	0.2430
Respiratory Failure[1]			
Baseline (−75 to −15)	40/11,773	Ref	-
Clearance (−14 to −1)	2/2,702	0.22 (0.03-1.64)	0.0523
0	0/193	0.00 (0.00-Inf)	0.9946
1-21	20/3,962	1.72 (0.42-6.98)	0.3181
22-42	29/3,793	3.37 (0.59-19.24)	0.0722
Baseline (−75 to −15)	40/11,773	Ref	-
Clearance (−14 to −1)	2/2,702	0.19 (0.03-1.40)	0.0322
0	0/193	0.00 (0.00-Inf)	0.9944
1-42	49/7,755	1.57 (0.40-6.15)	0.3987
Rheumatoid Arthritis and Polyarthritis			
Baseline (−75 to −15)	66/12,261	Ref	-
Clearance (−14 to −1)	8/2,814	0.53 (0.20-1.38)	0.0879

Interval in Days	Events/Person days	Incidence Rate Ratio (99% CI)	*p*
0	1/201	0.92 (0.07-12.41)	0.9376
1-42	47/8,331	1.05 (0.64-1.72)	0.7924
43-90	53/9,271	1.07 (0.67-1.73)	0.6993
Baseline (−75 to −15)	66/12,261	Ref	-
Clearance (−14 to −1)	8/2,814	0.53 (0.20-1.38)	0.0879
0	1/201	0.92 (0.07-12.41)	0.9376
1-90	100/17,602	1.06 (0.71-1.60)	0.6995
		Seizures	
Baseline (−75 to −15)	106/20,679	Ref	-
Clearance (−14 to −1)	18/4,746	0.74 (0.38-1.43)	0.2368
0s	1/339	0.57 (0.04-7.66)	0.5819
1-6	13/2,027	1.25 (0.59-2.67)	0.4498
		Stroke (Haemorrhagic)	
Baseline (−75 to −15)	26/9,150	Ref	-
Clearance (−14 to −1)	3/2,100	0.50 (0.10-2.42)	0.2594
0	0/150	0.00 (0.00-Inf)	0.9953
**1-7**	**11/1,042**	**3.82 (1.51-9.67)**	**0.0002**
**8-42**	**35/4,981**	**2.82 (1.42-5.60)**	**0.0001**
Baseline (−75 to −15)	26/9,150	Ref	-
Clearance (−14 to −1)	3/2,100	0.50 (0.10-2.42)	0.2594
0	0/150	0.00 (0.00-Inf)	0.9952
**1-42**	**46/6,023**	**3.03 (1.58-5.79)**	**0.0000**
		Stroke (Ischemic)	
Baseline (−75 to −15)	150/33,855	Ref	-
**Clearance (−14 to −1)**	**8/7,770**	**0.23 (0.09-0.59)**	**0.0001**
0	1/555	0.41 (0.03-5.39)	0.3696
1-7	9/3,878	0.52 (0.22-1.27)	0.0587
8-42	100/19,167	1.18 (0.84-1.64)	0.2075
Baseline (−75 to −15)	150/33,855	Ref	-
**Clearance (−14 to −1)**	**8/7,770**	**0.23 (0.09-0.59)**	**0.0001**
0	1/555	0.41 (0.03-5.39)	0.3696

Interval in Days	Events/Person days	Incidence Rate Ratio (99% CI)	*p*
1-42	109/23,045	1.07 (0.77-1.48)	0.6099
		Thrombocytopenia	
Baseline (−75 to −15)	33/5,185	Ref	-
Clearance (−14 to −1)	3/1,190	0.39 (0.08-1.86)	0.1205
0	0/85	0.00 (0.00-Inf)	0.9954
1-21	8/1,744	0.70 (0.25-1.95)	0.3679
22-42	16/1,719	1.43 (0.64-3.16)	0.2511
Baseline (−75 to −15)	33/5,185	Ref	-
Clearance (−14 to −1)	3/1,190	0.39 (0.08-1.86)	0.1205
0	0/85	0.00 (0.00-Inf)	0.9953
1-42	24/3,463	1.06 (0.53-2.13)	0.8326
		Transient Ischaemic Attack	
Baseline (−75 to −15)	46/10,736	Ref	-
Clearance (−14 to −1)	9/2,464	0.85 (0.33-2.18)	0.6614
0	0/176	0.00 (0.00-Inf)	0.9942
1-42	37/7,392	1.17 (0.66-2.06)	0.4813

[1]A temporal trend of 28 days was included to adjust for seasonal variation in respiratory failure incidence rates.

Bold values indicate statistically significant values where $p<0.01$.
